# Cardiac Imaging in Women with Ischemic Heart Disease

**DOI:** 10.3390/life13061389

**Published:** 2023-06-14

**Authors:** Amalia Peix

**Affiliations:** Institute of Cardiology and Cardiovascular Surgery, 17 No. 702, Vedado, Havana CP 10 400, Cuba; atpeix@gmail.com; Tel.: +53-53798428

**Keywords:** women, ischemic heart disease, cardiac imaging techniques, multimodality

## Abstract

Cardiac diseases are the main cause of death for both sexes worldwide. Treatment varies widely according to the sex of a patient, as there are differences in physiopathology, epidemiology, clinical presentation and management. However, women have been largely excluded from research studies in this field. At present, differences are starting to be recognized and more attention is being paid to the identification of female-specific (or emergent) atherosclerotic risk factors. Diagnostic testing also merits attention because cardiac imaging offers important information to help diagnosis and guide cardiac disease management. In this sense, multimodal imaging should be used with the most cost-effective approach, integrating this information into the clinical sphere according to the pretest probability of the disease. In this review, we address sex-specific features of ischemic heart disease that should be considered in the clinical assessment of women, as well as the value of different imaging techniques (including technical and clinical aspects) for management of women with ischemic heart disease, and identify future areas of action concerning ischemic heart disease in women.

## 1. Introduction

Cardiovascular disease (CVD) is the leading cause of mortality worldwide, in both women and men. According to the Lancet Women and Cardiovascular Disease Commission, published in 2021 [1], cardiac disease represents 35% of all deaths in women worldwide, and 275 million women were diagnosed with CVD in 2019. However, as has been stated in this report, CVD in women is understudied, under-recognized, underdiagnosed, and undertreated, and women are still under-represented in clinical trials. 

Thus, it is crucial to raise awareness of the importance of this health problem, as well as to consider of atherosclerotic risk factors -RFs- (both traditional and emergent female-specific) and the utilization of appropriate evidence-based guidelines with a female-specific approach in clinical practice.

In this review, the following topics are addressed: sex-specific features of ischemic heart disease (IHD) that should be considered in the clinical assessment of women with suspected or known IHD; the value of different imaging techniques (including technical and clinical aspects) for management of women with IHD, and future areas of action concerning IHD in women. 

## 2. Specific Features of Ischemic Heart Disease That Should Be Considered in the Clinical Assessment of Women with Suspected or Known IHD

### 2.1. Physiopathologic and Anatomic Differences

Compared to men, women show particular characteristics of IHD: there are physiopathologic differences, such as distal thrombus, plaque erosion, impaired coronary vasomotor function and microvascular dysfunction as main mechanisms explaining the development of IHD in women, contrary to plaque rupture in men [2]. Thus, women have less obstructive and extensive epicardial artery disease than men. Anatomically, women have smaller epicardial coronary arteries than men, which may complicate the precise evaluation of distal coronary arteries by coronary computed tomographic angiography (CCTA) [3]. In addition, women have thinner myocardial walls, which makes the assessment of non-transmural ischemia by cardiac magnetic resonance imaging (CMR) more difficult. Population-based studies have shown a lower calcium score [4] and atheroma volume [5] in women when compared to men. Women exhibit higher coronary blood flow at both rest and peak stress, but similar coronary flow reserve (CFR) [6].

### 2.2. Risk Factors

Although there are traditional atherosclerotic RFs present in both women and men, such as high blood pressure, diabetes mellitus (DM), dyslipidemia, smoking habits, obesity, physical inactivity, and family history of premature atherosclerosis, some atherosclerotic RFs present a higher risk in women than in men. For instance, DM confers a 45% higher risk of IHD [7], while smoking represents a 25% higher risk [8], and obesity a 64% higher risk [9] in women compared to men. Menopause is a traditional female-specific RF, which can be associated with higher levels of triglycerides, LDL-cholesterol and lower levels of HDL-cholesterol. Nevertheless, women are less likely to achieve lipid goals during treatment [10], mainly because they are not as aggressively treated as men.

Among the so-called emergent RFs, gynecologic and obstetric conditions merit special attention. Polycystic ovarian syndrome (PCOS) comprises hormonal and metabolic abnormalities such as impaired glucose tolerance, type 2 DM, vascular disease, dyslipidemia, and obstructive sleep apnea [11,12]. Early menopause is an independent risk factor for CVD, increasing risk by approximately 1.3-fold [13].

A detailed obstetric history should be registered for every woman being evaluated for a suspected IHD. Adverse pregnancy outcomes, such as hypertensive disorders, gestational diabetes, preterm delivery, multiparity (especially among women with five or more live births), and delivery of a small for gestational age infant, are now recognized as emerging risk factors for premature CVD in women [14,15,16,17]. A shared pathogenesis combining placental insufficiency and the development of a pro-inflammatory and antiangiogenic milieu has been suggested to explain the increased risk of developing a CVD in these patients [18].

Women with gestational diabetes have a higher CVD risk (twofold for stroke and fourfold for myocardial infarction (MI)) [19]. Regarding hypertensive disorders during pregnancy, Benschop et al. [20] reported that previously preeclamptic women have more modifiable cardiovascular RFs and develop coronary calcium approximately five years earlier, from the age of 45 years onwards, compared to women with normal blood pressure during pregnancy.

Other conditions that are not specific to women, but are more prevalent in women should also be considered, such as autoimmune diseases and depression/emotional stress.

Psychological stress is still an understudied and underappreciated cardiovascular RF that needs a tailored approach, particularly in women. The INTERHEART study showed the relationship between stress, depression and a woman’s first MI [21]. Of note, the negative effects of stress and depression after an MI are more common in younger women compared to men and older patients [22,23]. Moreover, after MI young women have a twofold likelihood of developing mental stress-induced MI compared to men (22% vs. 11%, *p* = 0.009) [24]. 

A final case that should be considered is cardiac damage post-radiotherapy in the case of thoracic irradiation in women with breast cancer or lymphoma, with symptoms that develop decades after radiotherapy. In this case, two mechanisms can explain the coronary artery disease (CAD) [25]: a macrovascular injury that accelerates age-related atherosclerosis, or a microvascular injury that reduces myocardial capillary density and causes a reduction of collateral flow/vascular reserve resulting in myocardial ischemia, as well as an increased capillary permeability of the pericardium via thickening and adhesions.

### 2.3. Clinical Differences

From a clinical point of view [26], more women than men have a sudden cardiac death as a first manifestation of the disease. In addition, more women than men die during the first year after an MI and after a coronary artery bypass grafting (CABG), and younger women exhibit higher mortality rates than men. 

Regarding the somewhat different clinical picture, it is important to consider that women’s ischemic symptoms are more often precipitated by mental or emotional stress than by physical stress. Symptoms have been considered “atypical”, because rather frequently women do not complain of an anginal chest pain, but of epigastric discomfort, as well as nausea, dyspnea and fatigue. In fact, these symptoms previously considered atypical have begun more recently to be contemplated as typical in women with IHD.

In addition, treatment following guideline-directed medical therapy and reperfusion strategies, either percutaneous coronary interventions (PCI) or CABG, are less applied in women compared to men [27].

## 3. Value of Different Imaging Techniques for Management of Women with IHD

The 2021 AHA/ACC/ASE/CHEST/SAEM/SCCT/SCMR Guideline for the Evaluation and Diagnosis of Chest Pain made recommendations for a focus on the uniqueness of chest pain in women [28]. In this sense, the guideline considers the following as recommendation class I, level of evidence B-NR [28]:

“Women who present with chest pain are at risk of underdiagnosis, and potential cardiac causes should always be considered.

In women presenting with chest pain, it is recommended to obtain a history that emphasizes accompanying symptoms that are more common in women with acute coronary syndromes”.

Considering the aforementioned pathophysiologic differences of IHD in women compared to men, as well as predominant plaque erosion, impaired coronary vasomotor function and microvascular dysfunction, it is crucial to identify in which scenarios the clinician should distinguish between women and men for an appropriate cardiovascular imaging approach [29,30]. In this sense, although CCTA shows sensitivity and specificity with respect to detecting coronary stenosis and coronary dissections, its diagnostic accuracy can be limited in women due to the smaller diameter of vessels [31]. On the contrary, CMR and positron emission tomography (PET) are valid options to evaluate microvascular dysfunction thanks to their capacity to measure myocardial blood flow (MBF) and CFR.

### Diagnostic Algorithm for IHD in Women Considering the Pretest Probability of the Disease in a Multimodal Cardiac Imaging Approach

In case of suspected stable IHD, several tables for calculations of pretest probabilities of presenting an obstructive CAD in symptomatic patients have been applied. The 2021 ACC guideline [28] includes one table that is particularly useful because, in addition to chest pain characteristics, age and sex, it considers calcium scoring, which allows for refining the calculation according to the amount of coronary calcium, mainly in the case of patients with intermediate-high pretest probabilities.

Thus, in women with a suspected IHD, the first step should be to calculate the pretest probability of having an obstructive CAD. Accordingly, selection of the diagnostic test should also take into account the availability of tests, local expertise and patient characteristics and preferences (recommendation class I, level of evidence C, according to the 2019 ESC Guidelines for the Diagnosis and Management of Chronic Coronary Syndromes) [28].

According to the pretest probability of the disease:

- In patients with a very low probability of obstructive CAD, diagnostic tests can be deferred, although according to clinical evaluation, other options may be considered [32], such as: an exercise test (without imaging) if the electrocardiogram (ECG) at rest is interpretable and the patient can exercise (although this is less useful for women, considering the lower sensitivity of the test compared to men and the possibility of false positives), or a coronary calcium scoring for a better refinement of risk (if not included in the pretest probability analysis).
- In patients with a low to intermediate probability and considering that CCTA has a very high negative predictive value, an anatomic approach with CCTA is the most effective option, independent of sex.
- In those with an intermediate to high probability, an ischemia-provoking test with imaging should be considered: stress-echocardiography, nuclear (either single-photon emission computed tomography -SPECT- or PET), or stress CMR. The type of applied stress will depend on functional capacity, ECG at rest and the type of test selected.
- In those patients with a very high pretest probability, there is no doubt that invasive coronary angiography (preferably with fractional flow reserve measurement) is the most effective option to choose, independent of sex.

If an obstructive CAD is diagnosed, an appropriate management strategy should be applied that takes into consideration the extent and severity of ischemia and anatomic characteristics of coronaries, independent of sex. The possibility of a microvascular dysfunction and other causes of chest pain should also be considered.

According to the 2019 ESC Guidelines for the Diagnosis and Management of Chronic Coronary Syndromes [33], a non-invasive functional imaging test for myocardial ischemia or CCTA is recommended as the initial test to diagnose CAD in symptomatic patients in whom obstructive CAD cannot be excluded by clinical assessment alone (recommendation class I, level of evidence B).

Table 1 shows a comparison of advantages and disadvantages of the different imaging techniques.

The use of solid-state cadmium zinc telluride (CZT) cameras has allowed for improved spatial resolution and increased camera sensitivity that has allowed for a lower required dose of the radiotracer and therefore, lower radiation exposure. The use of hybrid imaging (SPECT/CT) allows for attenuated-corrected images.

## 4. Echo-Stress 

Stress-induced wall motion abnormalities shown in echocardiography are an accurate indicator of IHD risk in women [34,35]. In a group of 14 diagnostic studies reviewed, Geleinjse et al. [35] found that pooled data showed nearly identical values for sensitivity and specificity in women and men using dobutamine stress echo (DSE). In seven of these studies, the diagnostic accuracy of DSE could be compared, reporting the same sensitivity (77%) in women and men. Specificity was 81% in women and 77% in men [35]. 

Echo-stress is a valid option to detect IHD in women with intermediate to high pretest probability of the disease, with diagnostic and prognostic advantages over stress tests without imaging. In experienced groups, echo-stress shows comparable or better specificity and lower costs compared to nuclear MPI [36,37], with the advantages of broader availability and no radiation exposure. However, echo-stress sensitivity is affected by the extent and location of CAD, being lower in patients with single-vessel disease [38]. Although dipyridamole stress echocardiography might have a role in assessments of microvascular disease [39,40], CFR measurement of echocardiography is still in development and not widely applied.

## 5. Nuclear Imaging Techniques

### 5.1. SPECT

SPECT myocardial perfusion imaging (MPI) is a widely available and validated technique for the detection of stress-induced ischemia. However, some problems should be taken into account and potential solutions implemented:

- In obese women there may be reduced specificity due to attenuation from large or dense breasts. In this case, the use of supine and prone/upright acquisitions [41,42] and, mainly, attenuation correction with hybrid SPECT-CT scanners if available, is indicated to solve this issue.
- Regarding the concerns of radiation dosage derived from the test, it is important to consider that new advances in software and gamma camera hardware, with iterative reconstruction, depth resolution recovery and noise reduction software, as well as the use of solid-state cadmium zinc telluride (CZT) cameras, have allowed SPECT to achieve a lower required dose and lower radiation exposure (6–9 mSv for ^99m^Technetium -^99m^Tc- low dose rest stress SPECT MPI) [43,44], with increased spatial resolution and sensitivity. In addition, the use of a stress-only protocol in cases with low to intermediate probability of an obstructive CAD and normal stress results also reduces radiation exposure, with only 3 mSv [45].

PET MPI is an accepted alternative to reduce radiation exposure if available. Rest-stress PET MPI studies with ^82^Rubidium (^82^Rb) or ^13^Ammonia (^13^NH3) represent a radiation dose of 2–3 mSv, especially with the use of 3D reconstruction [46].

In both SPECT and PET MPI, non-perfusion variables (reduced left ventricular ejection fraction -LVEF- or new wall motion abnormalities on the post-stress gated SPECT images indicating stunned ischemic myocardium, as well as transient ischemic dilation of LV) should also be considered, even with normal stress perfusion scans, as high risk features suggestive of obstructive CAD [47]. 

Another aspect to take into account is the functional response to the physical stress test, because electrocardiographic, chronotropic and blood pressure responses to exercise as well as the functional capacity constitute other non-nuclear variables that may suggest the presence of ischemia. A high-risk Duke treadmill score may suggest the presence of obstructive CAD with balanced ischemia [48]. 

### 5.2. PET and Myocardial Blood Flow (MBF)

Measurements of MBF and CFR comparing stress and rest values results are particularly useful in assessing the possibility of balanced ischemia in cases with normal perfusion, but also an intermediate to high probability of having an obstructive CAD and non-perfusion ancillary suggestive of ischemia. Additionally, in cases of microvascular dysfunction, which are particularly frequent in women, CFR is an important diagnostic tool [49]. 

In recent years, ^18^F-flurpiridaz has emerged as a promising tracer for PET-MPI evaluation of MBF [50]. In addition, ^18^F-flurpiridaz-PET-MPI presents higher specificity than SPECT-MPI for the detection of CAD in women [51], most likely related to the diagnostic performances of ^18^F-flurpiridaz- PET-MPI in patients with small ventricles [52].

On the other hand, vascular ^18^F-sodium fluoride PET (^18^F-NaF PET) has shown its value in documenting the early stages of plaque microcalcification [51] and to predict the progression of coronary plaques [53]. It is of interest that the intensity of ^18^F-NaF uptake in atherosclerotic plaques of patients without CAD is lower in women than in men [54], consistent with the lower burden of calcified plaque.

Dynamic SPECT with CZT cameras shows promise in the assessment of myocardial flow reserve and several papers have been published on the subject with acceptable results [55,56], but it may require more study for further validation before being accepted for routine use.

Hybrid imaging, both in cases of SPECT-CT and PET-CT, offers the possibility of evaluating coronary calcium with a very low radiation dose, which may be useful when the myocardial perfusion is normal, particularly when the degree of calcium is high. On the contrary, a 0-calcium score represents a very good prognosis. The prognostic value of coronary calcium is independent of sex, although men tend to have higher calcium scores for each vessel and higher mean total calcium scores compared to women [57].

## 6. Cardiac Magnetic Resonance (CMR)

CMR, in addition to the absence of ionizing radiation, allows for the quantitative evaluation of global and regional ventricular functions (gold standard tests), myocardial perfusion and MBF, including the ability to identify microvascular dysfunction, the possibility of tissue characterization with the identification of myocardial scar/viable tissue, and a comprehensive evaluation to exclude other cardiac causes of chest pain [58]. Of importance to this study is that there are no reported sex differences in diagnostic performance [59]. 

The suggested protocol [58] should include at least cine images at rest for evaluations of cardiac structures and functions; stress first-pass perfusion imaging (post-vasodilator stress with adenosine, dipyridamole or regadenoson); and T1-weighted images for evaluations of late gadolinium enhancements to assess scar/fibrosis.

## 7. Coronary Computed Tomography Angiography

**Figure 1:**
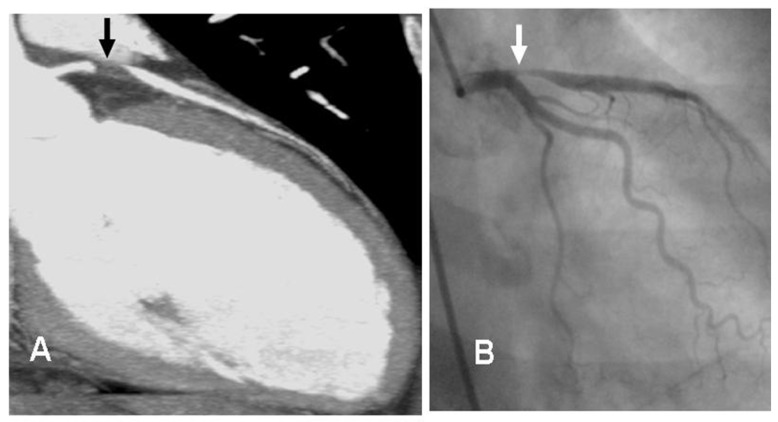
Comparison between CCTA and invasive angiography. Legend: Female, 52 y.o. who presents to the emergency room complaining of chest pain (not typical). At the ECG: ST elevation in precordial leads. Image (**A**) shows the stenosis of proximal segment of the left anterior descendent coronary artery (LAD), 99% of stenosis (black arrow). The patient was sent to the Interventional Cardiology Department (see invasive angiography in image (**B**)), the LAD lesion was confirmed (white arrow), and a stent was implanted. ECG: electrocardiogram. Image courtesy of Dr. Luis R. Llerena Rojas, Institute of Cardiology, Havana, Cuba.

Shaw et al. [60] reported that obstructive CAD was less prevalent in women than men (46% vs. 72%) and mortality was related to the number of stenosed vessels. Due mainly to the smaller diameter of coronary arteries in women, in spite of the overall high diagnostic accuracy for the detection of stable CAD, the sensitivity and specificity of CCTA is slightly lower in women than in men [41,61]. Figure 1 shows an example of a female patient with an acute coronary syndrome (ACS) and a 99% stenosis of the left anterior descendent artery on the CCTA.

Of interest to this study is the fact that CCTA findings are prognostic in men as well as in women [62], although with some differences: women display less high-risk plaque features than men [63], but when they appear they constitute a stronger predictor of MACE in women than in men [64].

## 8. Coronary Microvascular Dysfunction 

Middle-aged women have higher resting coronary blood flow compared to men [65,66], most likely related to differences in autonomic function. This may explain the presence of lower CFR values in women compared to men in invasive studies [67,68], although noninvasive studies did not find significant differences according to sex [69]. 

On the other hand, premenopausal women exhibit nearly 2-fold better coronary blood flow response than postmenopausal women and age-matched men [70]. This is most likely explained by the relationship between estradiol and the production of nitric oxide and estradiol receptor mediated vasodilation. Thus, the decrease of estrogen in postmenopausal women, in addition to low-grade systemic inflammation and increased sympathetic activity more prevalent in women, contribute to the development of coronary microvascular dysfunction (CMD) [71].

Pepine et al. reported that nearly 39% of the patients selected for coronary angiography because of suspected angina and/or positive stress test have non-obstructive CAD. This was more frequent in women than in men (approximately 50–70%) [72].

Two main presentations should be addressed: ischemia with no obstructive coronary arteries (INOCA), and myocardial infarction with no obstructive coronary arteries (MINOCA).

### 8.1. INOCA

In INOCA, the mismatch between blood supply and myocardial oxygen demands may be caused by CMD and/or epicardial coronary artery spasms [71,73]. Several algorithms have been published to assess patients with INOCA. Kunadian et al. presented an EAPCI Expert Consensus Document on INOCA, which addresses this diagnosis in a comprehensive way [74]. First of all, a clinical history considering RFs, previous events of ischemia and a physical examination should be taken in addition to an ECG.

Non-invasive diagnostic tests constitute the first-line tools, preferably those able to provide information about the coronary function, such as echocardiography (Doppler or myocardial contrast echo), cardiac PET or CMR. The choice will depend on availability, local expertise and clinical decision. The 2021 Guideline for the Evaluation and Diagnosis of Chest Pain [28] gives a class IIa recommendation for patients with INOCA to consider stress PET MPI and stress CMR with myocardial blood flow reserve measurements to diagnose CMD. If anatomical information on atherosclerosis is desired or there is a low clinical probability of CAD, a CCTA can be a valuable option.

**Figure 2:**
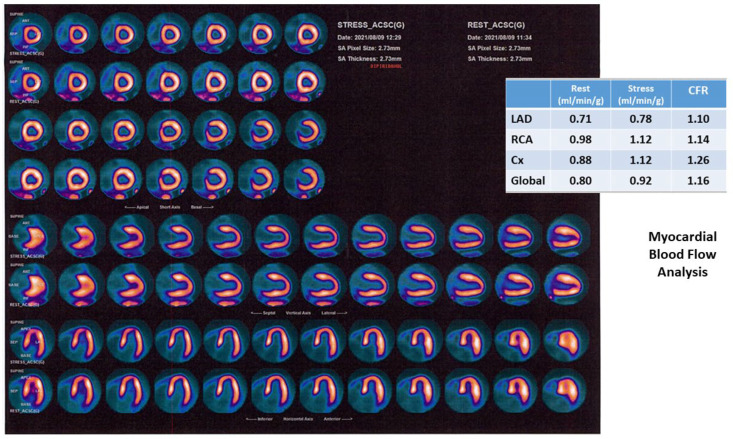
PET MPI in a patient with microvascular dysfunction. Legend: Female, 66 y.o., history of HBP, dyslipidemia, ex-smoker, early menopause. History of chest pain with several hospital admissions. Epicardial coronary arteries without lesions on invasive angiography. A PET study with stress/rest protocol with dipyridamole (0.56 mg/kg) was performed. Stress/rest images show a normal perfusion. Stress LVEF: 52%. Myocardial blood flow analysis shows a significant reduction of CFR, mainly in the LAD territory. Compatible with a microvascular dysfunction. HBP: high blood pressure; PET: positron emission tomography; LVEF: left ventricular ejection fraction; CFR: coronary flow reserve; LAD: left anterior descendent coronary artery; RCA: right coronary artery; Cx: left circumflex coronary artery. Image courtesy of Dr. Roberto Agüero, Fundación Centro Diagnóstico Nuclear, Buenos Aires, Argentina.

Figure 2 shows a case of a female patient with multiple RFs and history of chest pain with several hospital admissions. The invasive angiography did not show lesions on epicardial coronary arteries. A PET study rest/stress test with dipyridamole was performed and the CFR was reduced in all the territories, compatible with a microvascular dysfunction.

In patients with suspected CMD who are symptomatic but whose epicardial coronary arteries are angiographically normal or have <50% stenosis with preserved fractional flow reserve (FFR), or with suspected vasospasm, it is necessary to perform invasive functional tests to measure CFR, index of microvascular resistance (IMR), hyperemic myocardial velocity resistance index (HMR), and assess vasoreactivity (recommendation class IIa, level of evidence: B-NR, according to the 2021 Guideline for the Evaluation and Diagnosis of Chest Pain) [28]. This will allow for classifying INOCA patients into different phenotypes: epicardial vasospastic angina, microvascular angina or microvascular and epicardial vasospastic angina [74]. Standardized diagnostic criteria have been proposed by the Coronary Vasomotor Disorders International Study (COVADIS) Group for both mechanisms of INOCA: CMD and coronary vasospasm [75,76].

**Figure 3:**
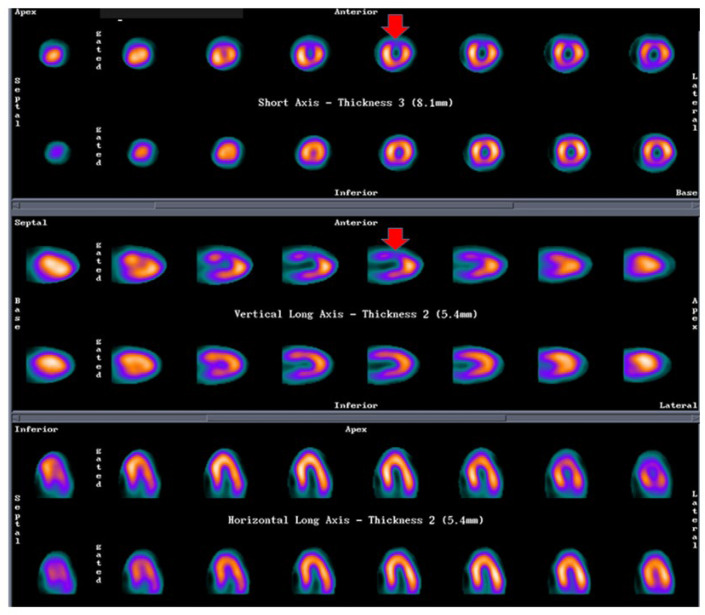
SPECT MPI in a patient with mental stress induced ischemia. Legend: Female, 57 y.o., postmenopausal, HBP. Angina with epicardial coronary arteries without lesions. Stress ECG: 1.5 mm ST depression, V1–V4. SPECT-MPI: Physical stress-induced ischemia (concordant with mental stress-induced ischemia). First line of each projection shows mental stress images, second line shows rest images. Compatible with anterior ischemia. This patient also had endothelial dysfunction measured as abnormal flow-mediated vasodilation in brachial artery with ultrasound. HBP: high blood pressure; ECG: electrocardiogram; SPECT: single photon emission tomography; MPI: myocardial perfusion imaging.

Mental stress-induced myocardial ischemia can be a common manifestation of INOCA in women [24,77], which can be diagnosed by a reduction of LVEF, new regional wall motion abnormalities or a perfusion defect in response to mental stress. In a small group of postmenopausal women with typical angina and normal coronary angiography studied via ^99m^Tc methoxy-isobutyl-isonitrile SPECT MPI (exercise stress/rest/mental stress protocol) and brachial artery endothelial function measurement via ultrasonography, we found a physical stress-induced ischemia (concordant with mental stress-induced ischemia, see Figure 3) associated with post-stress LVEF reduction and endothelial dysfunction (abnormal flow-mediated vasodilation) in 37% of patients [78]. 

### 8.2. MINOCA

MINOCA, more prevalent in women [79], is defined as an acute MI (as per the fourth universal definition) [80], with no obstructive coronary arteries on invasive coronary angiography, and no specific differential diagnosis, which requires excluding myocarditis and Takotsubo syndrome [81]. MINOCA may not have a known origin in 8–25% of cases [82], but can also be caused by coronary spasms and spontaneous coronary artery dissections. 

The treatment of MINOCA requires an individualised approach depending on the underlying diagnosis. 

At present it is recommended that patients with MINOCA secondary to plaque disruption or with evidence of ischemic damage on CMR receive dual antiplatelet therapy (12 months of treatment followed by a lifelong single agent), high-dose statin (including in patients with minimal plaque burden), β-blocker and renin-angiotensin-aldosterone system inhibitors/angiotensin II receptor blockers [83].

If there is an underlying diagnosis of epicardial or microvascular vasospasm, patients should receive calcium channel blockers, although nitrates and potassium channel activators may be considered as adjuncts in addition to renin-angiotensin-aldosterone system inhibitors/angiotensin II receptor blockers, and statin therapy may be considered if coronary atherosclerosis is identified [84]. An important differential diagnosis is Takotsubo syndrome (TTS), Takotsubo cardiomyopathy, stress-induced cardiomyopathy, or apical ballooning syndrome, because it can mimic an ACS. TTS is an acute and transient heart failure syndrome initially described by Sato et al. in 1991 [85], that predominantly occurs in post-menopausal women, though it is reported that up to 10% of TTS cases are male. Stressor events (emotional, physical or combined) usually trigger the clinical onset, but in about one-third of cases there was no preceding trigger [86]. Of note, according to the InterTAK diagnostic criteria [87], neurological disorders (subarachnoid hemorrhage, stroke/transient ischemic attack, or seizures) as well as pheochromocytoma may also serve as triggers. In addition, significant CAD does not constitute a contradiction in TTS.

**Figure 4:**
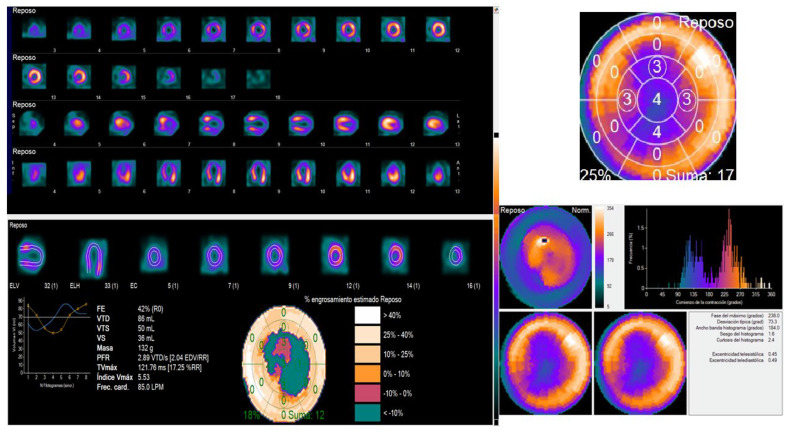
SPECT MPI in a patient with Takotsubo syndrome. Female, 63 y.o., admitted for angina during the last hour. ECG: ST elevation V2-V6, troponin 761 ng/mL. Epicardial coronary arteries without lesions. SPECT-MPI: shows a perfusion defect in all the apical segments (rest study), with an intraventricular desynchrony by phase analysis (Phase SD: 73 degrees and histogram bandwidth 184 degrees), as well as wall abnormalities also in apical segments, with a LVEF slightly reduced (42%) at rest. ECG: electrocardiogram; SPECT: single photon emission tomography; MPI: myocardial perfusion imaging; SD: standard deviation; LVEF: left ventricular ejection fraction.

**Figure 5:**
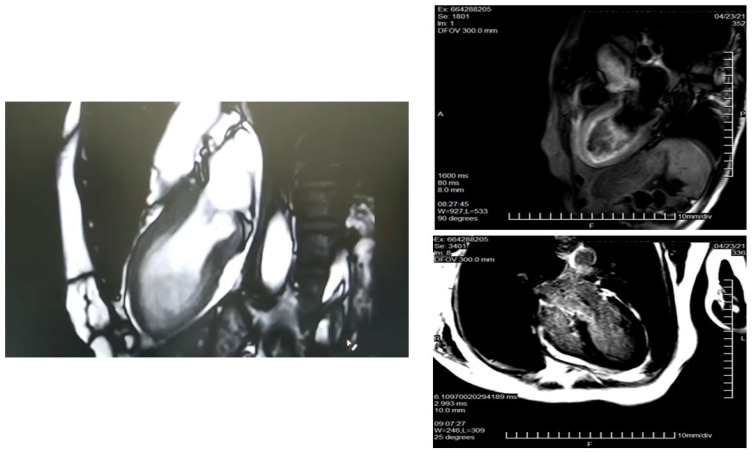
CMR in a patient with Takotsubo syndrome. Legend: Same patient as Figure 4. CMR study. Cine images with an apical ballooning. T2-weighted images showing myocardial oedema. There is no LGE. CMR: cardiac magnetic resonance; LGE: late gadolinium enhancement.

Multimodal non-invasive imaging is helpful for establishing the diagnosis, guiding therapy, and stratifying prognosis of TTS patients in both the acute and post-acute phase [86]:

- Echocardiography constitutes the first approach, particularly in the acute care setting. It allows the evaluation of ventricular functions (both systolic and diastolic) in the acute and the recovery phases, the identification of the ballooning pattern (mainly apical-midventricular) and the circumferential pattern of wall motion abnormalities, as well as the early detection of complications.
- CMR provides a more comprehensive picture of cardiac morphology and function, and offers the possibility of tissue characterization: typically, the presence of a reversible tissue injury (oedema) and the absence of irreversible tissue injury (LGE), contributing to the differential diagnosis with MI and myocarditis.
- CCTA may be an option instead of invasive coronary angiography in cases of stable patients with low suspicion of ACS; patients with a history of TTS and suspected recurrence; critical clinical conditions usually associated with TTS (e.g., sepsis, subarachnoid hemorrhage, or ischemic stroke) or where invasive coronary angiography could cause complications due to the patient’s condition.
- Although the role of nuclear imaging in TTS has not yet been well established in clinical practice, assessments of myocardial perfusion, adrenergic innervation and metabolic activity may help in the diagnosis [88,89]. For instance, if there is normalized LV wall motion, the delayed recovery of glucose metabolism (by FDG-PET) and sympathetic innervation (by ^123^I-MIBG scintigraphy) may allow for the diagnosis of TTS in patients with delayed presentation. Although the coronary microcirculation is transiently compromised in TTS, its physiological role is still unclear. A reduction of perfusion tracer counts as a result of regional myocardial wall thinning at the apex, due to both artefacts and partial volume effects, which may mimic ACS, has been reported [86]. Figure 4 (SPECT-MPI) and Figure 5 (CMR) present the case of a patient with TTS.

Regarding the management of Takotsubo patients, the following aspects should be considered [90]:

- Treatment requires inpatient care with cardiology services and is largely supportive until LV function spontaneously returns, usually within 21 days of onset.
- In stable patients, diuretics and vasodilators can be used for pulmonary congestion. Angiotensin-converting enzyme inhibitors, angiotensin II receptor blockers, and/or beta-blockers are used to reduce patient workload and control hypertension. Aldosterone receptor antagonists or angiotensin receptor-neprilysin inhibitors may be beneficial.
- For patients with unstable hemodynamics, inotropes should not be used if there is a LV outflow tract obstruction (LVOTO). Beta-blockers and intravenous fluids are appropriate. If LVOTO is not present, use inotropes and vasopressors or LV assist device if needed.
- Anticoagulation should be initiated in patients with large areas of cardiac hypokinesis to reduce risk of major cerebral or vascular events.

## 9. Future Areas of Action Concerning IHD in Women

In order to achieve an adequate clinical decision, the appropriate management of IHD in women requires more basic clinical research in order for clinicians to better understand the physiopathologic mechanisms and differences according to a patient’s sex. In addition, a more detailed assessment should be made of the particularities of atherosclerotic risk factors in women, the influence of hormones, inflammation, pregnancy, and factors such as the mental stress and its triggering properties.

Other important aspects to address are the socioeconomic disparities in cardiovascular risk factors and outcomes present in women, particularly those of minority racial or ethnic backgrounds. Inadequate access to effective contraception, postpartum follow-up, and maternity leave, in addition to reduced access to care, low income and lack of social support can lead to excess rates of myocardial infarction, stroke, and cardiovascular death in at-risk women later in life. To overcome these disparities, policy changes, education and training, investments in sex-specific science and health policy advocacy, empowerment of patient-centered care through community-based solutions, as well as innovations in health care delivery and more diversity in inclusion of patients in clinical trials, are very much needed.

## 10. Conclusions

This review highlights sex-specific considerations useful for choosing the most appropriate cardiac imaging modality in a scenario of multimodal imaging techniques in women with IHD, with particular focus on the challenges and opportunities of contemporary management. Several future areas of action concerning IHD in women are also presented.

## Figures and Tables

**Table 1:** Advantages and disadvantages of imaging tests.

Imaging Test	Advantages	Disadvantages
Stress echo	No radiation, high availability, lower costs	Poor acoustic windows, less reproducible
SPECT MPI	Good reproducibility, validated for ischemia detection. Uses stress-only protocols if possible	Radiation exposure, need for attenuation correction for anterior defects in women
PET MPI	Validated for ischemia detection, MBF and CFR can be measured	Radiation exposure, although less than SPECT MPI
CMR	Validated for ischemia detection, MBF and CFR can be measured. Standard measurement for ventricular function. No radiation	Less availability, higher costs, claustrophobia, patients with implantable cardiac devices
CCTA	High negative predictive value. Measurement of calcium score in addition to coronary anatomy	Radiation exposure

SPECT: single photon emission computed tomography; PET: positron emission tomography; MPI: myocardial perfusion imaging; CMR: cardiac magnetic resonance; CCTA: cardiac computed tomography angiography. References [6,32].

## Data Availability

Not applicable.
